# 改进的QuEChERS法结合超高效液相色谱-串联质谱技术测定土壤、沉积物和地表水中26种除草剂残留

**DOI:** 10.3724/SP.J.1123.2025.02016

**Published:** 2025-12-08

**Authors:** Li ZHAO, Lin MA, Lanqi HUANG, Jianbo CHEN, Weifang ZHU

**Affiliations:** 1.上海市农业技术推广服务中心，农业农村部农药质量检验测试中心（上海），上海 201103; 1. Shanghai Agriculture Technology Extension & Service Centre，the Ministry of Agriculture and Rural Affairs Pesticide Quality Inspection and Testing Center （Shanghai），Shanghai 201103，China; 2.上海市青浦区农产品质量安全中心，上海 201700; 2. Shanghai Qingpu District Agricultural Product Quality and Safety Center，Shanghai 201700，China

**Keywords:** 超高效液相色谱-串联质谱, QuEChERS, 除草剂, 土壤, 沉积物, 地表水, ultra-high performance liquid chromatography-tandem mass spectrometry （UHPLC-MS/MS）, QuEChERS, herbicide, soil, sediment, surface water

## Abstract

为有效监测环境样品中除草剂残留水平并提供环境安全评估技术依据，建立了QuEChERS前处理结合超高效液相色谱-串联质谱（UHPLC-MS/MS）技术同时测定土壤、沉积物及地表水中26种除草剂的分析方法。土壤与沉积物样品经乙腈振荡提取、盐析后采用QuEChERS法净化；地表水经乙腈提取后无需净化直接进样分析。实验优化了仪器检测和前处理条件，考察了方法的线性关系、基质效应、检出限和定量限。在0.1~50 μg/L范围内线性关系良好，相关系数（*r* ^2^）均大于0.999 0。26种除草剂在土壤、沉积物和地表水中的基质效应为-35.2%~14.6%。土壤和沉积物中26种除草剂的定量限为0.5 μg/kg，地表水中26种除草剂的定量限为0.1 μg/L。三水平加标试验中：土壤和沉积物的添加水平分别为0.5、1.0和10.0 μg/kg，回收率为73%~108%和73%~102%，RSD为4.5%~16.2%和3.8%~19.7%；地表水添加水平为0.1、1.0和10.0 μg/L，回收率为74%~110%，RSD为4.0%~15.0%。将该方法用于分析上海市6个蔬菜种植区周边环境样本中26种除草剂的污染状况，结果显示，土壤基质中扑草净、异丙甲草胺及甲嘧磺隆阳性率较高，检出率分别为52.9%、52.9%、29.4%，含量范围为0.8~490.4 μg/kg、0.5~219.8 μg/kg和1.0~562.6 μg/kg；沉积物中扑草净检出率达83.3%，含量为1.5~6.7 μg/kg；地表水中甲嘧磺隆、扑草净和西草净均有检出，极限浓度值分别为12、2.5和1.1 μg/L。研究建立的方法简单、快速、准确、稳定，实用性强，可用于检测土壤、沉积物和地表水中的26种除草剂残留，为监测除草剂的残留污染和环境行为提供参考。

## Abstract (en)

As chemical agents that selectively inhibit weed growth， herbicides play a crucial role in enhancing crop yields. With increasing weed resistance， the environmental residue problems caused by excessive application have become increasingly prominent. Studies indicate that only 20%–30% of field-applied herbicides are effectively utilized， with the remainder entering environmental media such as the atmosphere， soil， sediment， and surface water through runoff and leaching. Recent frequent occurrences of vegetable phytotoxicity incidents in Shanghai have been traced to potential associations with herbicide residues in surface water， further highlighting the urgent need to establish multi-residue analytical methods for environmental media. An analytical method was established based on ultra-high performance liquid chromatography-tandem mass spectrometry （UHPLC-MS/MS） for determining 26 herbicide residues in soil， sediment， and surface water. Instrumental detection parameters were optimized， including electrospray ionization mode， mobile phase， and chromatographic column. The mobile phase consisted of 0.1% formic acid aqueous solution （A） and acetonitrile （B） with the following gradient elution program： 0–0.5 min， 2%B； 0.5–1 min， 2%B–50%B； 1–4 min， 50%B–65%B； 4–6 min， 65%B–75%B； 6–8 min，75%B–85%B； 8–10 min， 85%B–95% B； 10–11 min， 95%B. Soil and sediment samples were extracted via acetonitrile oscillation followed by salting-out and purified using the QuEChERS method. Surface water samples were directly analyzed after acetonitrile extraction without purification. Different amounts of purification agents were investigated during sample pretreatment. Calibration curves were established by plotting the relationship between analyte concentration and measured peak area using pure solvent and matrix-matched standards. All 26 herbicides showed good linearity in the range of 0.1–50 μg/L with correlation coefficients exceeding 0.999 0. Matrix effects ranged from -35.2% to 14.6% across different matrices. Limits of quantification （LOQs） were 0.5 μg/kg for soil and sediment， 0.1 μg/L for water samples. The herbicides were spiked into soil and sediment at spiked levels of 0.5， 1， and 10 μg/kg， and into surface water at 0.1， 1， and 10 μg/L， respectively. The average recoveries for the 26 herbicides in soil， sediment， and surface water were in the ranges of 73%–108%， 73%–102%， and 74%–110%， respectively. The RSDs for the 26 herbicides were in the ranges of 4.5%–16.2%， 3.8%–19.7%， and 4.0%–15.0%， respectively. The developed method was applied to analyze the contamination status of the 26 herbicides in environmental samples collected from six vegetable cultivation zones in Shanghai. Results revealed distinct pollution patterns： In soil matrices， prometryn （PMT）， metolachlor （MTA）， and sulfometuron-methyl （SMTM） showed higher detection rates of 52.9%， 52.9%， and 29.4%， respectively， with content ranges of 0.8–490.4 μg/kg， 0.5–219.8 μg/kg， and 1.0–562.6 μg/kg. Sediment samples exhibited an 83.3% detection rate for PMT （1.5–6.7 μg/kg）. In surface water， SMTM， PMT， and simetryne （STN） were detected with maximum contents of 12， 2.5 and 1.1 μg/L， respectively， indicating differential migration behaviors across environmental compartments. The proposed method is simple， rapid， accurate， stable， and highly practical. It can be used to detect the 26 herbicide residues in soil， sediment， and surface water and provides a reference for monitoring the residual pollution and environmental behavior of herbicides.

除草剂作为选择性抑制杂草生长的化学制剂，在提升农作物产量方面发挥着重要作用，然而，随着杂草抗性增强，其过量施用导致的生态环境残留问题日益凸显^［1-3］^。研究显示，田间施用的除草剂仅20%~30%被有效利用，其余部分通过径流、淋溶等途径进入大气、土壤、沉积物和地表水等环境介质^［4］^。近年，我国多地区环境样本中均有典型除草剂残留检出，纪丙鑫等^［5］^报道苏州生态涵养区地表水与沉积物中扑草净含量高达0.75 µg/L，证实其对水生生态系统存在潜在风险；王蔚菁团队^［6］^在兴凯湖水体中检出阿特拉津等5种除草剂，最高含量达6.09 µg/L。值得注意的是，环境介质中除草剂残留水平不仅受土壤理化特性、气候条件及农艺管理措施等多因素影响，更与区域种植结构呈现显著空间关联性^［7，8］^。现有针对生态环境残留问题的研究多聚焦地表水与沉积物^［9，10］^，农田耕作土壤的除草剂残留特征尚未被揭示。本研究将土壤-沉积物-地表水作为完整环境体系进行研究，旨在系统探究除草剂的迁移归趋规律。

环境样品中除草剂检测主要依赖色谱及其联用技术，包括高效液相色谱（HPLC）^［11］^、液相色谱-串联质谱（LC-MS/MS）^［12-14］^和气相色谱-串联质谱（GC-MS/MS）^［15，16］^。其中，LC-MS/MS因兼具高灵敏度与选择性，已成为农残分析的主流平台。在样品前处理方面，固相萃取法^［11，13，16，17］^虽净化效果好但操作烦琐，液-液分配法^［14，15］^则存在有机溶剂消耗量大等缺陷。相比之下，QuEChERS方法^［12，18］^凭借其快速、低成本及可扩展性等优势，在复杂基质处理中展现出独特适用性。本研究基于前期对上海市56处典型农田的实地调研结果，结合历史药害事件中除草剂的检出情况，运用QuEChERS前处理与超高效液相色谱-串联质谱（UHPLC-MS/MS）技术，成功建立了土壤、沉积物及地表水中26种除草剂的多残留同步检测方法。通过系统优化提取净化流程与仪器参数，实现了不同基质中目标物的高效富集与精准定量。该方法显著提升了检测通量，为农田环境介质除草剂残留监测及生态风险预警提供了可靠技术支撑。

## 1 实验部分

### 1.1 仪器、试剂与材料

1290-6460超高效液相色谱-三重四极杆质谱仪（美国Agilent公司）；Milli-Q超纯水仪（美国Millipore公司）；AB104-S电子天平（梅特勒-托利多国际贸易上海有限公司）；RML-80 Pro长轴混匀仪（群安实验仪器有限公司）。

乙腈、甲醇（色谱纯，德国Merck公司）。甲酸、醋酸铵（色谱纯，上海安谱实验科技股份有限公司）。C_18_、PSA、GCB、无水MgSO_4_（美国Agilent公司）。0.22 μm水相针式滤膜、0.22 μm有机相针式滤膜、定性滤纸（上海安谱实验科技股份有限公司）。26种除草剂标准品（纯度≥97%，德国Dr. Ehrenstorfer公司）。

实验所用土壤样本、沉积物样本及地表水样本均采集于上海市市郊蔬菜种植区周边自然环境。

### 1.2 标准溶液配制

用甲醇分别配制26种目标除草剂的单标储备液（1 000 mg/L），置于棕色小瓶中于-20 ℃保存。用甲醇稀释标准储备液得到10 mg/L的混合标准工作液，置于棕色小瓶中于4 ℃冷藏保存。使用前，用甲醇或空白环境样本基质溶液稀释混合标准溶液，配成0.1、0.5、1、10、50 μg/L系列浓度的标准工作溶液。

### 1.3 样品前处理

#### 1.3.1 提取

土壤/沉积物：称取样品10 g（精确至0.01 g）于50 mL离心管中，再加入20 mL乙腈，振荡提取30 min后抽滤，滤液转移至装有5 g氯化钠的50 mL离心管中，剧烈振荡约1 min，静置约0.5 h，在4 000 r/min下离心4 min，待净化。

地表水：将地表水样用滤纸过滤后移取10 mL于50 mL离心管中，加入10 mL乙腈剧烈振荡提取1 min，加入5 g氯化钠，剧烈振荡1 min，静置约0.5 h，在4 000 r/min下离心4 min，待净化。

#### 1.3.2 净化

土壤/沉积物：移取2 mL待净化液加入到装有20 mg C_18_和300 mg无水MgSO_4 _的15 mL离心管中，涡旋1 min后。在4 000 r/min下离心4 min。收集全部上清液，过0.22 μm有机相滤膜，待检测。

地表水：取2 mL上清液，过0.22 μm水相滤膜，待检测。

### 1.4 仪器条件

Agilent ZORBAX SB-C_18_色谱柱（150 mm×2.1 mm，5 μm）；柱温30 ℃；流动相A：0.1%甲酸水溶液，流动相B：乙腈；梯度洗脱程序：0~0.5 min，2%B；0.5~1 min，2%B~50%B；1~4 min，50%B~65%B；4~6 min，65%B~75%B；6~8 min，75%B~85%B；8~10 min，85%B~95%B；10~11 min，95%B。流速：0.45 mL/min，进样量：2 µL。

电喷雾离子源，正离子扫描模式；检测方式为多反应监测（MRM）模式，鞘气温度：350 ℃；鞘气流量：12 L/min；雾化气压力：275.8 kPa；毛细管电压：3 500 V；干燥气温度：300 ℃；干燥气流量：11 L/min。其他质谱采集参数见表1。

**表 1:** 26种除草剂的保留时间和质谱采集参数

No.	Pesticide	Retention time/min	Precursor ion （*m/z*）	Qualitative ion pair （*m/z*）	Fragmentor/V	Collision energies/eV
1	metamitron （MTT， 苯嗪草酮）	5.10	203.1	175.0^*^， 104.0	120	15， 15
2	bensulfuron-methyl （BSFM， 苄嘧磺隆）	5.45	411.0	149.0^*^， 182.1	120	25， 25
3	nicosulfuron （NSF， 烟嘧磺隆）	5.45	411.0	213.0^*^， 182.0	100	15， 15
4	thifensulfuron-methyl （TFSFM， 噻吩磺隆）	6.02	388.0	205.0^*^， 167.0	120	20， 10
5	metsulfuron-methyl （MSFM， 甲磺隆）	6.19	382.1	167.0^*^， 141.0	120	15， 15
6	chlorsulfuron （CSF， 氯磺隆）	6.47	358.0	167.0^*^， 141.0	100	15， 15
7	sulfometuron-methyl （SMTM， 甲嘧磺隆）	6.49	365.2	150.0^*^， 107.0	120	15， 15
8	ethametsulfuron-methyl （EMSFM， 胺苯磺隆）	6.54	411.0	196.0^*^， 168.0	120	15， 15
9	simetryne （STN， 西草净）	6.95	214.1	144.0^*^， 124.0	120	20， 20
10	penoxsulam （POS， 五氟磺草胺）	7.07	484.0	195.1^*^， 164.0	120	35， 35
11	atrazine （ATZ， 莠去津）	7.41	216.2	174.1^*^， 96.1	120	15， 20
12	tribenuron Methyl （TBM， 苯磺隆）	7.61	396.1	181.0^*^， 155.1	120	20， 20
13	ametryn （AMT， 莠灭净）	8.00	228.1	186.1^*^， 96.1	120	20， 25
14	pyrazosulfuron （PRSF， 吡嘧磺隆）	8.25	415.2	182.0^*^， 139.1	100	15， 15
15	chlorimuron-ethyl （CRE， 氯嘧磺隆）	8.39	415.1	185.9^*^， 120.7	140	20， 35
16	mefenacet （MFA， 苯噻酰草胺）	8.96	299.2	148.0^*^， 120.0	120	25， 25
17	prometryn （PMT， 扑草净）	9.14	242.2	158.1^*^， 200.2	120	20， 20
18	terbutryn （TBT， 特丁净）	9.31	242.1	186.0^*^， 71.0	120	15， 20
19	acetochlor （ATC， 乙草胺）	10.00	270.1	224.0^*^， 148.0	80	10， 10
20	metolachlor （MTA， 异丙甲草胺）	10.03	284.0	252.0^*^， 176.0	110	10， 5
21	metamifop （MTF， 恶唑酰草胺）	11.36	441.1	123.0^*^， 288.0	120	30， 25
22	pinoxaden （POA， 唑啉草酯）	11.91	401.0	317.2^*^， 57.2	120	25， 25
23	pretilachlor （PTA， 丙草胺）	11.93	312.0	252.0^*^， 176.0	120	15， 30
24	clethodim （CTD， 烯草酮）	12.18	360.1	268.2^*^， 164.0	120	5， 20
25	butachlor （BTA， 丁草胺）	12.73	312.0	238.0^*^， 162.0	120	15， 10
26	pendimethalin （PDT， 二甲戊灵）	12.86	282.1	212.0^*^， 194.0	100	9， 17

* Quantitative ion.

## 2 结果与讨论

### 2.1 仪器条件优化

#### 2.1.1 质谱条件优化

采用正离子扫描模式，对26种除草剂的单标准溶液进行一级全扫描，得到稳定的母离子，然后进行二级质谱扫描，找到两个响应信号稳定的子离子作为定性子离子，其中响应信号较强的子离子作为定量子离子，并优化得到每种除草剂的母离子和子离子所需的最佳碎裂电压和碰撞能量。最后，结合基质空白和基质标准液的离子扫描图，进一步优化参数，确定26种除草剂在MRM模式下的质谱采集参数，见表1。

#### 2.1.2 液相色谱条件优化

以甲醇-水（50∶50，体积比）为流动相，比较了ZORBAX SB-C_18_（150 mm×2.1 mm，5 μm）、Poroshell 120 EC-C_18_（150 mm×3.0 mm，2.7 μm）和ZORBAX Eclipse Plus C_18_ RRHD（150 mm×2.1 mm，1.8 μm）色谱柱对26种除草剂混合标样的分离情况。结果表明，所有化合物在3种型号色谱柱上均有较好的保留行为，但在ZORBAX SB-C_18_柱上响应最高，故选择该色谱柱。

在此基础上，实验还考察了不同流动相体系对目标化合物色谱峰形和灵敏度的影响。以甲醇作为有机相，水相选择不同浓度的醋酸铵溶液（2、5 mmol/L）和不同体积分数（0.05%、0.1%、0.2%）的甲酸水溶液分别进行实验。结果表明，流动相中酸的添加能明显改善色谱峰形，并能提高离子化效率，而醋酸铵的添加对化合物的质谱响应值影响不大。目标化合物灵敏度与甲酸含量呈正相关，当甲酸体积分数为0.1%时，目标化合物响应最好，甲酸体积分数增加至0.2%时，目标化合物响应无明显变化。因此，选择含有0.1%甲酸溶液作为水相。确定水相后，实验对比了有机相为甲醇或乙腈对目标化合物的保留能力以及峰形的影响。甲醇的保留能力较乙腈稍强，但有机相为甲醇时，五氟磺草胺、苯噻酰草胺、特丁净响应信号明显低于乙腈。最终选择含有0.1%甲酸水溶液-乙腈作为流动相。

综上，在优化的仪器条件下，26种除草剂的总离子流图见图1。

**图1:**
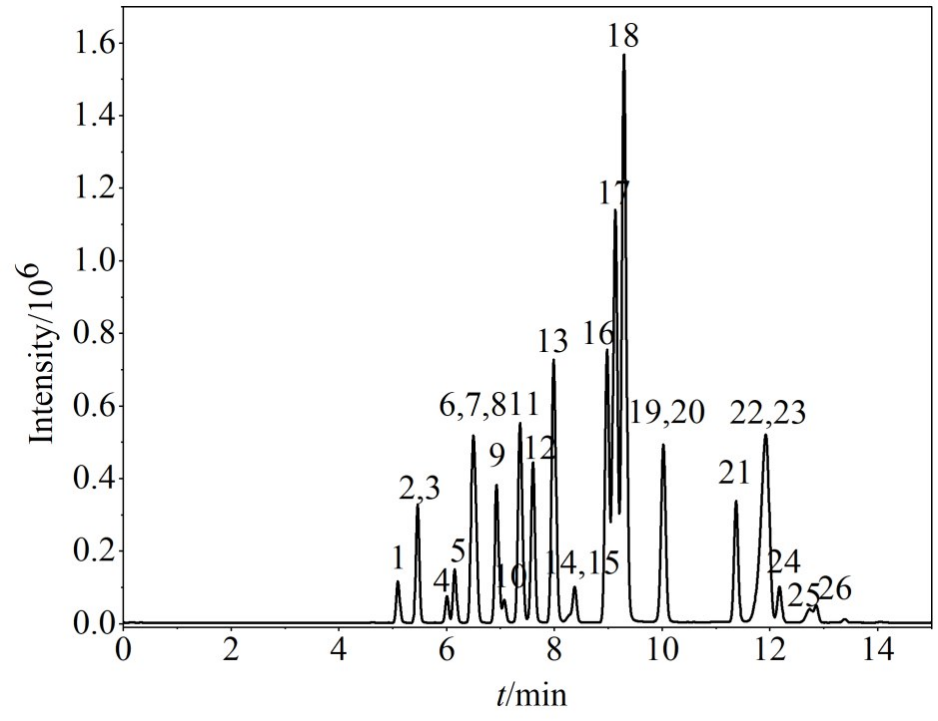
26种农药（10 μg/L）混合标准溶液的总离子流图

### 2.2 样品前处理条件优化

#### 2.2.1 提取剂的选择

基于QuEChERS方法的高通量特性，本研究优先采用该技术进行前处理以提升提取效率。环境土壤及沉积物基质中含腐殖酸、色素、脂类等复杂干扰组分，可能影响目标化合物色谱行为并加速仪器损耗。因此，优化提取溶剂对平衡提取效率与基质净化效果具有决定性作用。当前农残分析中常用萃取溶剂包括乙酸乙酯、乙腈、二氯甲烷及丙酮等。本研究通过溶剂极性、安全性、基质兼容性评估筛选最优方案：丙酮和乙酸乙酯作为提取剂时，会提取出更多的共萃物，对化合物的离子化抑制更强；二氯甲烷虽萃取范围广，但毒性较高且易乳化；相比之下，乙腈通过蛋白质沉淀作用可大大减少样品基质中蜡质、脂肪和一些亲脂性色素的提取量，对各目标化合物均有相对较好的提取效率。因此，选择乙腈作为提取溶剂。

本研究选择10 μg/kg作为加标水平进行不同溶剂提取效能的评价，能更好匹配实际污染水平。本研究以土壤为基质系统评估了乙腈及其甲酸改性溶剂的提取性能（图2）。实验表明：通过添加体积分数为0.1%~5%的甲酸可显著增强质子化作用，改善目标物溶解性。当甲酸体积分数达1%时，26种目标物的平均回收率从采用乙腈的72%提升至82%。进一步增加酸度至5%时，回收率无显著变化。综合考虑提取效率及实验安全性，最终确立1%甲酸乙腈为最优提取溶剂，该方案下26种除草剂的平均回收率稳定在70%~112%。

**图2:**
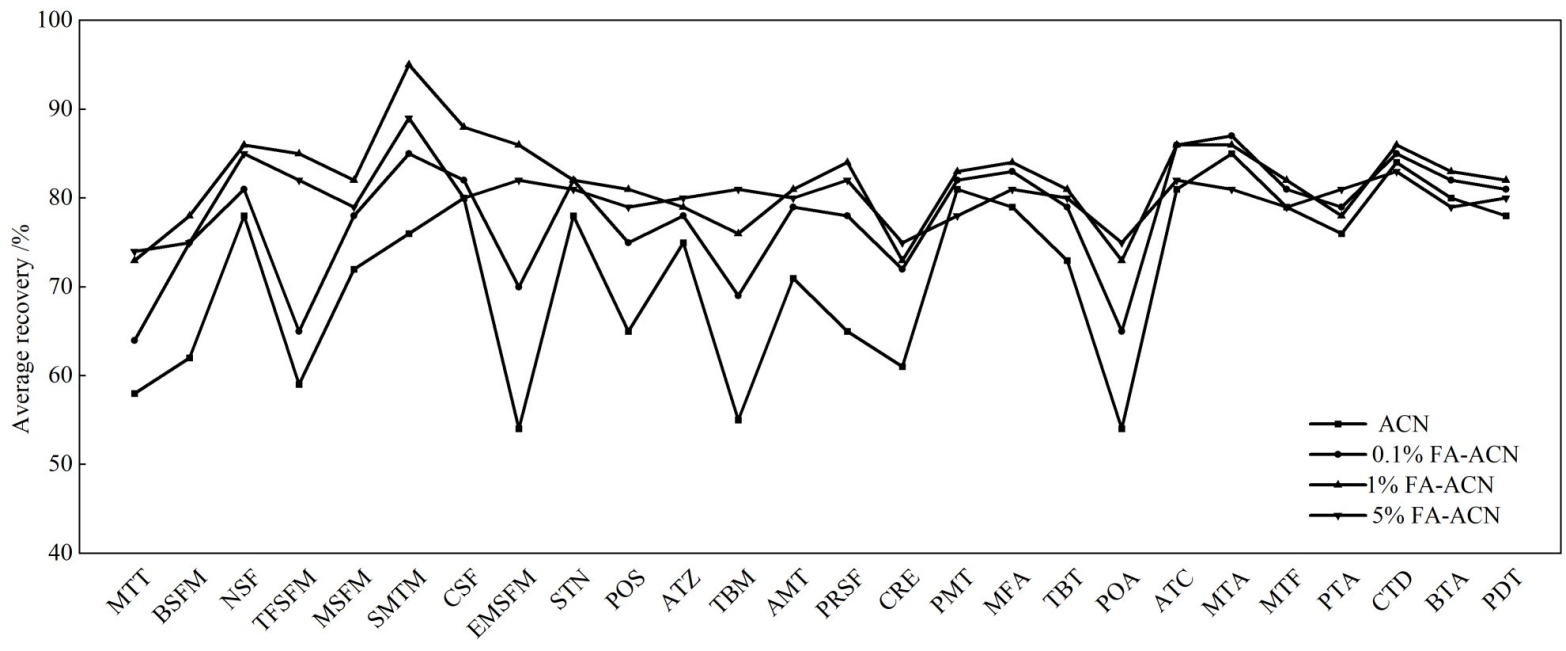
土壤中26 种除草剂（10 μg/kg）在不同提取溶剂中的平均回收率（*n*=5）

#### 2.2.2 净化剂的选择

本研究以AOAC 2007.01推荐吸附剂组合（C_18_、PSA、GCB、无水MgSO_4 _）为基础，针对农田环境基质特性进行定向优化。其中：C_18_通过疏水作用选择性吸附脂类与甾醇；PSA凭借氨基/乙二胺-*N*-丙基硅烷基团螯合金属离子并去除糖类衍生物；GCB通过*π*-*π*相互作用消除甾醇及平面结构色素，但可能对含苯环/杂环结构的农药（如烟嘧磺隆）产生竞争性吸附；无水MgSO₄则通过水合反应降低溶剂极性，提升相分离效率^［19-22］^。

为平衡净化效率与目标物回收率，本研究选择10 μg/kg作为加标水平，分别评估了C_18_、PSA及GCB的基质净化效果，每2 mL上清液用量分别为50、50、10 mg（图3）。结果显示：C_18_组综合性能最优，26种除草剂平均回收率在70%~120%，显著高于PSA组回收率；而GCB因强平面吸附特性导致烟嘧磺隆、苄嘧磺隆等双环磺酰脲类除草剂回收率低于60%，证实其不适用于含芳香杂环的目标体系。进一步优化C_18_用量，每2 mL上清液用量分别为20、50、100 mg。结果表明：20、50 mg剂量下目标物回收率无明显差异，平均回收率分别为93.7%、94.1%；当剂量提升至100 mg时，脂溶性较强除草剂（如丁草胺）回收率明显下降，可能因为过量C_18_对中等极性农药的非特异性吸附。基于经济性与效能平衡，最终确定C_18_最佳用量为每2 mL上清液20 mg。最终确定每2 mL上清液添加20 mg C_18_为最优净化体系，该方案下26种除草剂的平均回收率为72%~116%。

**图3:**
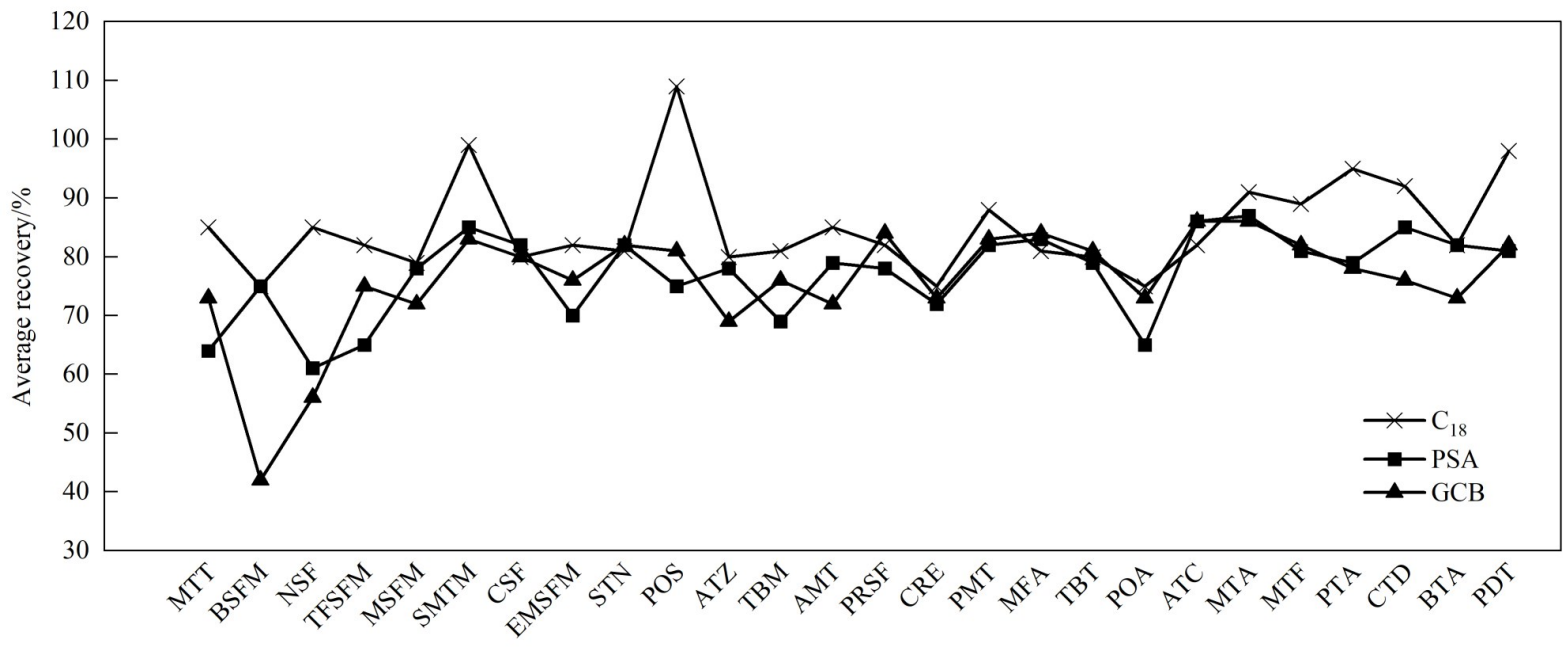
土壤中26种除草剂（10 μg/kg）在不同净化剂下的平均回收率（*n*=5）

### 2.3 方法评价

#### 2.3.1 基质效应影响

在土壤、沉积物等复杂环境样品的分析中，基质效应会影响离子的电离程度，进而对目标化合物的定量结果造成影响^［23］^。称取空白土壤、沉积物和地表水样，按照1.3节和1.4节进行前处理和检测分析，确定其不含26种除草剂，再用空白样品溶液和甲醇溶剂配制10 μg/kg的基质标准溶液和溶剂标准溶液，在完全相同的色谱条件下检测，根据两种检测液的色谱响应值按式（1）计算基质溶液的基质效应（ME）。


(1) $$ME=\frac{A_{m}-A_{s}}{A_{s}}\times 100\%$$

式中，*A*_m_：基质溶液中农药的色谱峰面积；*A*_s_：纯溶剂中农药的色谱峰面积。当|ME|<20%时为弱基质效应，可用溶剂标准溶液进行定量；当20%≤|ME|<50%时为中等强度基质效应，需采用基质匹配标准溶液进行定量；当|ME|≥50%时为强基质效应，需采用基质匹配标准溶液进行定量^［24，25］^。

26种除草剂在土壤、沉积物和地表水中的基质效应见表2。从表2可见，土壤、沉积物和地表水中26种除草剂的基质效应分别为‒35.2%~‒1.6%、‒30.6%~13.4%、‒19.2%~14.6%。在土壤和沉积物中，烟嘧磺隆和氯嘧磺隆呈现中等强度基质效应，其余24种除草剂呈弱基质效应；在地表水中，26种目标化合物均呈弱基质效应，因此，最终确定采用基质匹配标准曲线进行土壤和沉积物中目标化合物的定量分析，采用溶剂标准曲线进行地表水中目标化合物的定量分析。

**表 2:** 26种除草剂在土壤、沉积物和地表水中的基质效应

Pesticide	Matrix effects/%		
	Soil	Sediment	Surface water
MTT	-5.5	-6.2	2.2
BSFM	-9.3	-10.3	1.1
NSF	-35.2	-29.6	14.6
TFSFM	-14.5	-13.8	4.3
MSFM	-8.2	-10.8	3.2
SMTM	-15.6	-13.9	14.2
CSF	-17.3	-15.7	8.9
EMSFM	-12.8	13.4	7.4
STN	-8.2	-9.9	2.6
POS	-4.3	-5.4	1.6
ATZ	-6.7	-7.6	2.1
TBM	-11.6	-15.3	-19.2
AMT	-2.3	-3.9	2.8
PRSF	-8.9	-7.4	4.4
CRE	-25.8	-30.6	3.9
PMT	-3.2	-5.1	-2.5
MFA	-1.6	-2.9	4.5
TBT	-5.4	-8.6	1.5
POA	-8.8	-9.1	1.7
ATC	-6.1	-5.3	2.0
MTA	-1.9	-1.4	5.8
MTF	-4.4	-6.6	-2.1
PTA	-5.9	-5.5	9.7
CTD	-7.2	-9.7	1.9
BTA	-10.6	-12.3	8.7
PDT	-17.6	-18.1	6.2

#### 2.3.2 方法的线性、灵敏度、准确度和精密度

按照1.2节分别配制质量浓度为0.1~50 μg/L的26种除草剂土壤、沉积物混合基质标准溶液以及纯溶剂标准溶液。以各待测物的质量浓度为横坐标，对应峰面积为纵坐标绘制校准曲线。结果显示，在0.1~50 μg/L范围内，26种目标物化合物线性关系良好，相关系数（*r* ^2^）为0.999 0~0.999 9。根据GB/T 35655-2017要求，采用逐步稀释法计算检出限（LOD），采用加标回收法确定定量限（LOQ）。结果显示，26种目标物在土壤、沉积物和地表水中的LOD均为0.01 μg/kg；土壤和沉积物中LOQ为0.50 μg/kg，地表水中LOQ为0.1 μg/kg，满足欧盟2002/657/EC标准要求^［26］^。

通过三水平加标试验验证方法可靠性。土壤和沉积物空白样品加标水平为0.5、1.0、10. μg/kg；地表水空白样品加标水平为0.1、1.0、10. μg/L。按1.3节前处理方法进行加标回收试验，每个水平平行测定6次，计算平均回收率与相对标准偏差（RSD）。结果显示（表3），土壤基质中26种除草剂的回收率为73%~108%，RSD为4.5%~16.2%；沉积物基质中26种除草剂的回收率为73%~102%，RSD为3.8%~19.7%；地表水基质中26种除草剂的回收率为74%~110%，RSD为4.0%~15.0%。关键性能指标满足农业农村部2386号公告《农药残留检测方法国家标准编制指南》^［27］^中规定的农残检测要求（回收率60%~120%，RSD≤20%），证实该方法具备多基质兼容性与高通量检测潜力。

**表 3:** 26种除草剂在土壤、沉积物和地表水中的加标回收率和相对标准偏差（*n*=6）

Compound	Recoveries/% （RSDs/%）								
	Soil			Sediment			Surface water		
	10 μg/kg	1.0 μg/kg	0.5 μg/kg	10 μg/kg	1.0 μg/kg	0.5 μg/kg	10 μg/L	1.0 μg/L	0.1 μg/L
MTT	108 （8.3）	107 （7.6）	100 （15.3）	102 （14.3）	96 （15.7）	100 （16.4）	101 （12.1）	107 （7.2）	103 （11.9）
BSFM	106 （12.2）	103 （10.6）	96 （14.8）	97 （14.3）	96 （16.1）	97 （17.7）	98 （13.1）	110 （4.0）	98 （12.5）
NSF	86 （10.7）	87 （6.9）	83 （13.3）	86 （10.7）	84 （8.9）	87 （17.3）	88 （9.0）	98 （12.2）	87 （13.8）
TFSFM	88 （6.9）	87 （7.3）	84 （16.2）	87 （6.8）	84 （9.0）	84 （19.7）	89 （8.2）	95 （14.3）	86 （13.5）
MSFM	89 （6.4）	88 （5.9）	90 （9.3）	89 （5.8）	85 （7.9）	90 （9.3）	91 （5.4）	96 （13.4）	89 （9.6）
SMTM	77 （6.1）	78 （8.5）	81 （9.6）	80 （8.1）	78 （3.8）	84 （16.6）	83 （10.5）	103 （12.3）	84 （11.4）
CSF	81 （8.5）	81 （8.5）	79 （7.4）	82 （8.7）	80 （9.4）	84 （16.3）	87 （8.0）	87 （14.6）	85 （10.1）
EMSFM	73 （5.0）	77 （9.3）	73 （5.0）	78 （10.3）	74 （5.2）	80 （18.4）	79 （14.0）	82 （12.1）	79 （14.1）
STN	76 （7.1）	80 （8.4）	80 （13.2）	81 （8.4）	79 （8.9）	86 （17.8）	83 （10.9）	75 （5.0）	80 （13.2）
POS	79 （9.1）	78 （10.4）	82 （11.9）	82 （9.5）	79 （9.2）	88 （15.6）	81 （10.7）	74 （4.7）	88 （11.6）
ATZ	91 （4.6）	87 （10.8）	88 （8.2）	90 （4.9）	81 （11.3）	89 （14.2）	92 （4.3）	82 （13.6）	89 （9.8）
TBM	82 （10.1）	91 （13.7）	87 （8.1）	83 （10.0）	83 （11.3）	90 （13.8）	85 （10.9）	80 （8.8）	88 （9.9）
AMT	84 （9.4）	92 （12.2）	81 （8.5）	83 （7.9）	89 （14.5）	84 （15.4）	87 （10.3）	96 （15.0）	83 （11.7）
PRSF	85 （6.1）	85 （6.5）	84 （6.3）	86 （3.9）	81 （7.9）	86 （14.5）	87 （7.8）	95 （14.7）	89 （6.0）
CRE	84 （6.3）	82 （6.5）	88 （7.3）	84 （6.5）	79 （7.1）	89 （13.7）	88 （6.3）	83 （7.4）	88 （7.9）
PMT	84 （10.8）	87 （11.2）	88 （10.8）	84 （10.7）	80 （8.7）	88 （15.7）	89 （8.0）	97 （13.6）	86 （10.8）
MFA	78 （10.7）	77 （12.0）	81 （10.3）	78 （9.8）	75 （6.3）	82 （17.2）	85 （10.5）	80 （11.9）	82 （12.9）
TBT	76 （11.5）	89 （15.0）	76 （11.5）	73 （3.7）	82 （15.6）	79 （18.4）	83 （12.7）	95 （15.0）	79 （13.7）
POA	90 （13.8）	95 （14.2）	94 （13.5）	82 （12.3）	92 （17.5）	93 （16.4）	93 （12.3）	91 （14.5）	91 （13.5）
ATC	84 （4.5）	84 （4.5）	82 （5.8）	80 （8.9）	81 （6.1）	87 （13.1）	87 （6.1）	88 （13.6）	86 （8.4）
MTA	90 （12.2）	95 （10.1）	87 （8.7）	83 （13.2）	88 （15.2）	90 （13.6）	91 （9.9）	100 （13.0）	87 （8.2）
MTF	97 （13.6）	96 （8.7）	96 （11.4）	86 （14.2）	87 （11.8）	94 （13.7）	96 （11.5）	99 （11.0）	94 （9.5）
PTA	76 （10.6）	84 （15.0）	83 （11.9）	78 （8.4）	78 （12.3）	87 （16.6）	84 （10.7）	84 （13.6）	83 （11.9）
CTD	93 （11.1）	91 （8.7）	91 （10.0）	83 （10.7）	85 （11.8）	92 （14.7）	93 （9.8）	100 （11.3）	88 （7.2）
BTA	82 （11.8）	89 （11.3）	82 （11.8）	83 （10.4）	82 （11.0）	82 （16.8）	87 （9.7）	89 （10.8）	84 （14.1）
PDT	87 （5.9）	90 （8.4）	95 （16.1）	82 （9.3）	86 （12.7）	93 （17.6）	89 （6.5）	98 （13.6）	93 （14.8）

#### 2.3.3 方法应用

为验证方法实用性，本研究于2024年采集上海市郊6个蔬菜种植区周边环境样本27份（土壤17份、沉积物6份、地表水4份），采用本方法进行多残留检测，每个样本进行3次平行测定，定量结果平均值如表4所示，土壤基质中扑草净、异丙甲草胺及甲嘧磺隆检出率分别为52.9%、52.9%和29.4%，其中06号点位同时出现3种除草剂峰值含量（扑草净490 μg/kg、异丙甲草胺220 μg/kg、甲嘧磺隆563 μg/kg）；沉积物中扑草净检出率达83.3%（06点位含量30 μg/kg），为太湖流域沉积物背景值^［5］^的2倍；与苏州生态涵养区对比发现：上海农田土壤扑草净最大残留量（490 μg/kg）较苏州沉积物极限含量（21 μg/kg）^［5］^高23倍，这种量级差异可能与种植结构差异（蔬菜vs水稻）及施药制度（频次/剂量）密切相关。地表水中甲嘧磺隆、扑草净和西草净均有检出，最高含量分别为12 μg/L（02点位）、2.5 μg/L（06点位）和1.1 μg/L（04点位），我国《地表水环境质量标准》（GB 3838-2002）中暂未规定农药的最大允许污染浓度，参考2020年欧洲议会和欧盟理事会发布的新指令（EU）2020/2184^［28］^，天然水体中单一农药质量浓度不得超过0.1 μg/L，总质量浓度不得超过0.5 μg/L，本次监测结果超过欧盟标准，表明检测时间段内本地存在特异性污染源。值得注意的是，52%阳性样本检出2~4种除草剂共存，提示存在复合污染风险。此外，沉积物样本及地表水中甲嘧磺隆的检出证实其具有沉积物-水再悬浮扩散潜力，需结合水动力模型进一步开展长期归趋模拟，建议对重点区域除草剂使用建立清单制度，并依据本方法开展常态化监测。

**表 4:** 土壤和沉积物实际样品的检测结果（*n*=3）

Matrix	Monitoring site	Sample No.	Pesticide residues/（μg/kg）				
			SMTM	STN	PMT	MTA	PDT
Soil	S01	S01-soil-001	ND	ND	ND	ND	ND
		S01-soil-002	ND	ND	ND	ND	ND
	S02	S02-soil-001	ND	ND	ND	1.1	ND
		S02-soil-002	ND	ND	ND	ND	ND
	S03	S03-soil-001	8.4	ND	1.1	ND	ND
		S03-soil-002	ND	ND	0.8	1.6	5.9
		S03-soil-003	11.8	ND	ND	0.5	1.8
		S03-soil-004	1.0	ND	ND	0.5	9.8
	S04	S04-soil-001	ND	1.0	0.9	4.0	6.7
		S04-soil-002	ND	0.9	0.9	3.8	ND
		S04-soil-003	ND	ND	ND	ND	ND
	S05	S05-soil-001	ND	ND	ND	ND	ND
		S05-soil-002	ND	ND	0.8	ND	ND
	S06	S06-soil-001	ND	ND	1.5	219.8	ND
		S06-soil-002	220.4	ND	490.4	40.9	ND
		S06-soil-003	562.6	ND	2.7	1.0	ND
		S06-soil-004	ND	ND	0.8	ND	ND
Sediment	S01	S01-sediments-001	ND	ND	ND	ND	ND
	S02	S02-sediments-001	8.0	ND	1.5	ND	ND
	S04	S04-sediments-001	ND	3.0	2.1	ND	ND
	S05	S05-sediments-001	ND	ND	6.5	ND	11.0
	S06	S06-sediments-001	ND	ND	29.6	ND	ND
		S06-sediments-002	ND	ND	6.7	1.2	ND
Surface water	S01	S01-water-001	0.5^*^	ND	ND	ND	ND
	S02	S02-water-001	12.0^*^	ND	ND	ND	ND
	S04	S04-water-001	ND	1.1^*^	0.6^*^	ND	ND
	S06	S06-water-001	ND	ND	2.5^*^	ND	ND

ND： <LOQ. * Unit was μg/L.

## 3 结论

本研究以蔬菜种植区周边环境样本（土壤、沉积物和地表水）为基质，开展了26种除草剂残留分析方法研究，对仪器检测条件和前处理条件进行了考察。结果表明：建立的残留分析方法操作简单，方法稳定，定量准确，实用性强，能满足环境样品中26种除草剂的痕量残留分析，可为这26种除草剂在环境样品中的残留污染监测和环境行为实验提供参考方法。
